# EXERGAME INTERVENTION TO PROMOTE FAMILY CAREGIVERS’ SOCIAL SUPPORT, PHYSICAL ACTIVITY, AND WELL-BEING

**DOI:** 10.1093/geroni/igac059.3120

**Published:** 2022-12-20

**Authors:** Xin Yao Lin, Margie Lachman

**Affiliations:** Brandeis University, Waltham, Massachusetts, United States; Brandeis University, Waltham, Massachusetts, United States

## Abstract

Family caregivers often experience high stress, social isolation, poor mental and physical health, and have a sedentary lifestyle. The current study was a randomized trial (Nf76) comparing the effectiveness of Go&Grow (social vs non-social exergame app) to promote well-being through increased social support and physical activity for family caregivers over a 6-week intervention. Both groups received daily reminders to use the app. Findings showed the treatment group increased significantly more than the control group in well-being (management of distress) and social support (satisfaction with contact quality). There was an indirect effect of change in social support in which the treatment group increased more than the control group in satisfaction with contact quality, which in turn led to more positive affect and less loneliness. The increase in steps ranged from 3% to 12% across conditions. Social support moderated the relationship between condition and step changes, such that those in the treatment group who increased more in overall social support and knowing others’ experiences increased steps more than those with less support, while change in steps for the control group was not related to support level. In the treatment group, those who used the social features of the app more frequently had a greater increase in steps compared to those who used the social features less often. These findings can offer new insights into implementing behavior change mechanisms using exergames for sedentary and socially isolated family caregivers to improve their health with modifiable behavioral factors such as physical activity and social support.

